# Effects of a Grain Source (Corn Versus Barley) and Starter Protein Content on Performance, Ruminal Fermentation, and Blood Metabolites in Holstein Dairy Calves

**DOI:** 10.3390/ani10101722

**Published:** 2020-09-23

**Authors:** Mehdi Kazemi-Bonchenari, Mehdi Mirzaei, Mehdi HosseinYazdi, Mohammad Hossein Moradi, Mahdi Khodaei-Motlagh, Adel Pezeshki

**Affiliations:** 1 Department of Animal Science, Faculty Agriculture and Natural Resources, Arak University, Arak 38156-8-8349, Iran; mirzaee.1984@gmail.com (M.M.); mehdihoseinyazdi@yahoo.com (M.H.); h-moradi@araku.ac.ir (M.H.M.); mmotlagh2002@gmail.com (M.K.-M.); 2 Department of Animal and Food Sciences, Oklahoma State University, Stillwater 74078, OK, USA; adel.pezeshki@okstate.edu

**Keywords:** starch source, protein content, dairy calves, growth, amino acid, nitrogen efficiency

## Abstract

**Simple Summary:**

Dairy calf producers are concerned about feeding barley grain to dairy calves due to its rapid starch fermentation rate in the rumen compared to corn grain. Therefore, corn grain is still the main energy source in starter diets in dairy calves. However, some studies on bull and dairy calves indicated that there are some potentials for including barley grain in their diets with positively influencing the performance and feed efficiency. Optimum protein content in starter diet is an important factor influencing the animal growth response to grain source. This may be due to adequate nitrogen availability rate when starch is rapidly degraded in the rumen. Therefore, we hypothesized that dairy calves may produce different growth response to grain source with various ruminal starch degradation rate (high degradation rate in barley grain vs. low degradation rate in corn grain) when fed with starters with different protein content. Here we show that the average daily gain, feed efficiency, and ruminal fermentation profile were improved in dairy calves fed with barley grain. Furthermore, when dairy calves receive high protein content in their starter diet, barley grain improves growth performance in comparison with corn grain. Our study suggests that barley grain can be included in dairy calf starter diet when a starter with higher protein content is provided.

**Abstract:**

The effects of a grain source (corn grain (CG) vs. barley grain (BG)) and starter protein content (19% vs. 22% CP, dry matter basis) on growth performance, digestibility, ruminal fermentation, and blood metabolites were evaluated in Holstein dairy calves. Forty 3-day-old female calves with a starting body weight of 39.3 kg were subjected to four treatments in a completely randomized design with two by two factorial arrangements. Treatments were: (1) CG + 19% CP (CG-19CP); (2) CG + 22% CP (CG-22CP); (3) BG + 19% CP (BG-19CP); and (4) BG + 22% CP (BG-22CP). All calves were weaned at 59 days of age and remained in the study until 73 days of age. Starter and total DM intake were not affected by grain source and dietary protein content (*p* > 0.05). The average daily gain and feed efficiency were improved, and ruminal total short-chain fatty acid, propionate, and butyrate concentrations were increased in BG calves compared to CG calves (*p* < 0.05). The ruminal concentrations of ammonia nitrogen (d 71; *p* = 0.02) and acetate (d 35; *p* = 0.02) were increased in CG fed calves compared to BG. The greatest wither height (*p* = 0.03) and blood insulin concentration (*p* = 0.03) were seen in BG-22CP treatment. In conclusion, BG has marginal benefit in the height of calves when fed with diet containing 22% CP which may be recommendable in replacement heifer rearing programs.

## 1. Introduction

Grains are the main source of energy in dairy calves’ starter diet. The energy content of the grains is positively correlated with starch content [1]. There is a positive linear relationship between dietary starch content and dry matter digestibility, average daily gain, and growth performance [2]. Among different starch sources available as feedstuff in dairy calves’ diet, there is a tendency to feed calves with corn grain (CG) rather than other high-starch grains [3,4]. This is likely due to the slower starch degradation rate of CG compared to other high-starch grain sources such as barley grain (BG) which is related to various starch granule structures and shapes, as well as its coverage/linkage with protein or lipid components [5]. Processing of the grains increases the surface area exposure and improves ruminal and total tract starch digestibility [6,7]. Among different processing methods, grinding has been considered as a simple and less expensive processing method for grains [4]. The faster ruminal starch degradation rate of BG compared with CG when the ground grains are fed creates some concerns for inducing subacute ruminal acidosis [3]. This can be critical for dairy calves when they are fed with high-starch grains as they are more susceptible to ruminal pH drop than mature ruminants [8]. However, high levels of BG are commonly incorporated in rations of dairy cows [9], fattening bulls [10], and dairy calves [3,11]. Optimum dietary particle size [12], adequate level of forage in the diet [13,14], and proper fermentation rate of starch [15] are major factors influencing the feeding efficiency of high-starch grains in dairy calves.

Regardless the starch source and content in the starter feed of dairy calves, starter protein content and its degradability can also influence the animal growth response to feeding various high-starch grains. This is probably due to alteration the ruminal fermentation profile as well as changes in microbial protein yield reached into small intestine that can influence animal performance [4]. There are varied levels of starter protein content in studies that compared CG and BG in dairy calves such 23% in Khan et al. [3] and 20.5% by Mirzaei et al. [11], which can influence the *n* availability for ruminal microbes and eventually the animal response. Effects of starter protein content on growth performance of dairy calves have been evaluated extensively [16,17]. Different dietary protein concentrations provide animals with various levels of amino acids (AA) which has been shown to play a pivotal role in growth performance of pre-weaning ruminants [4,18]. The interactive effect between ruminal starch and nitrogen availabilities on ruminal fermentation and animal growth response has been well documented in dairy calves [19,20]. For instance, feeding starter protein source with different degradability (normal or extruded soybean meal) with different ruminal starch degradability rates (corn, barley, and dried whey) results in greater weight gain when a non-structural carbohydrate source was fed with less ruminal degradable protein source [19]. To the best of our knowledge, there is no published data assessing the interaction of starter’s grain source with protein content on growth performance of dairy calves. The hypothesis of the present study was that dairy calves may produce different responses to grain source with various ruminal starch degradation rate when fed starters with different protein content. Hence, the objective of the present study was to determine the effects of starter’s grain source and protein content on growth performance, nutrient digestibility, ruminal fermentation, and blood metabolites in Holstein dairy calves. 

## 2. Materials and Methods

### 2.1. Experimental Design, Treatments, and Animal Management

The present study was carried out at a commercial dairy farm (Baharan Farm, Isfahan, Iran). All animal procedures were approved by the Animal Care and Use Committee of Arak University (Protocol #IR2018011) following the guidelines outlined by Iranian Council of Animal Care (1995) [21]. A total of forty 3-day-old Holstein female dairy calves with initial BW of 39.3 ± 1.9 kg were randomly assigned to experimental diets (10 calves per treatment) in a 2 by 2 factorial arrangement with the factors of grain source (corn vs. barley) and starter protein content (19% vs. 22% CP, DM basis). Immediately after birth, calves were separated from their dams, weighed, and moved to individual pens (1.2 by 2.5 m) bedded with sand, which was renewed every 24 h. The animals were fed 5 L of colostrum at the first 12 h of life (2.5 L until one h after birth and 2.5 L at 12 h after the first feeding). Colostrum was fed for first 2 d of life. Calves received 4 L/d whole milk in buckets twice a day at 0900 and 1800 h from d 3 to 10, and 7 L/d from d 11 to 52 of the study followed by 3 L/d from d 53 to d 59 of the study. All calves were weaned on d 59 of the study and the experimental diets were continued until d 73 of study. Experimental treatments were starters with: (1) corn grain + 19% CP (CG-19CP); (2) corn grain + 22% CP (CG-22CP); (3) barley grain + 19% CP (BG-19CP); and (4) barley grain + 22% CP (BG-22CP). Experimental diets were formulated to meet the National Research Council (2001) nutrients requirements [22]. Ingredients and chemical composition of experimental diets are presented in Table 1. Corn and barley grains were ground using a hammer mill with a 3 mm screen size. Experimental diet particle size distribution, as well as their geometric mean particle sizes (GMPS), were measured by dry sieves (ASAE, 1995) [23]. Starter feed was fed ad libitum to permit at least 10% orts over a 24-h period. Starter feed refusals were collected and recorded at 0730 h and fresh starter feed was fed at 0800 h. The calves had free access to water throughout the experimental period. 

### 2.2. Growth Performance and Parameters

Individual body weight was recorded on days 3 (initial day), 17, 31, 45, 59 (weaning day), and 73 (final day) of the experiment. The average daily gain (ADG) and feed efficiency (BW gain/total DM intake) were also calculated. Growth parameters including heart girth, body length, body girth, wither height, hip height, and hip width were measured at the start of the experiment (d 3), weaning (d 59), and the last day of the experiment (d 73) according to the method described by Khan et al. [24] for dairy calves.

### 2.3. Diet, Fecal, Ruminal, and Blood Samples Collection

Feeds and orts samples were collected weekly. Fecal samples were collected via rectal palpation at 6, 12, and 18 h after the morning meal during 4 consecutive days (from d 68 to 72). Rumen fluid (30 mL) was collected (3–4 h after morning feeding) on d 35 (pre-weaning) and d 71 (post-weaning) of the experiment using a stomach tube fitted to a vacuum pump and its pH was measured immediately (HI 8318, Hanna Instruments, Cluj-Napoca, Romania). Ruminal fluid samples were squeezed through four layers of cheesecloth. An aliquot (10 mL) was preserved with 2 ml of 25% meta-phosphoric acid and frozen at −20 °C for later analysis. Blood samples were collected from the jugular vein into 10 mL dry tubes 4 h after morning feeding (at 1200 h) on d 36 (pre-weaning) and d 72 (post-weaning). The blood samples were transferred to the laboratory, centrifuged at 15,000× *g* for 20 min at +4 °C for collection of serum. The collected serum was stored at −20 °C until analysis.

### 2.4. Feed and Fecal Chemical Analysis and Digestibility Calculations

Feed and ort samples were dried in a convection oven (60 °C for 48 h). Subsamples of dried feeds and orts were mixed thoroughly and ground in a mill to pass a 1-mm screen before chemical analyses for CP (method 988.05; AOAC, 2002) and ether extract (method 920.39; AOAC, 2002) [25]. Starch content of experimental diets were calculated based on Cornell Net Carbohydrate and Protein System (CNCPS, v. 6.1). Calcium was determined by atomic absorption using spectrophotometer (Shimadzu, AA-670, Japan), and phosphorous was determined by atomic absorption using UV-spectrophotometer (Shimadzu, UV-2100, Japan). Diet neutral detergent fiber (NDF) was analyzed without sodium sulfite, but with the inclusion of α-amylase [26]. Dried fecal samples were analyzed for DM, NDF, CP, and ether extract to calculate apparent nutrient digestibility based on acid insoluble ash procedure (AIA) using the following formula: AD (%) = 100 − 100 × (MD/MF) × (NF/ND), where AD is the apparent digestibility (%), MD is the marker in the diet (%), MF is the marker in the feces (%), NF is the nutrient in the feces (%), and ND is the nutrient in the diet (%) [27]. 

### 2.5. Ruminal Fermentation Profile

After thawing the rumen fluid samples at room temperature, they were analyzed for short-chain fatty acids (SCFA) using gas chromatography (model CP-9002, Chrompack, Delft, the Netherlands) with a 50 m (0.32 mm ID) silica-fused column (CP-Wax Chrompack Capillary Column; Varian, Palo Alto, CA) as described previously [28]. For measuring the ruminal NH_3_-N concentration, ruminal fluid sub-samples were thawed at room temperature and clarified by centrifuging (15,000× *g* for 20 min at +4 °C). The clarified supernatant was then decanted and analyzed for NH_3_-N using a modified phenol-hypochlorite method adapted from Broderick and Kang [29]. 

### 2.6. Blood Biochemical Analysis

At the start of the experiment on d 3, blood serum total protein (TP) concentration of calves was measured to ensure that animals received adequate colostrum before initiating the experiment. Only calves with a blood TP concentration > 5.6 mg/dL were included in the study to assure that immune function of calves are not compromised prior to starting the study [4]. The mean values of TP for treatments were 6.19, 6.21, 6.15, and 6.23 mg/dL for CG-19CP, CG-22CP, BG-19CP, and BG-22CP, respectively. Serum subsamples (d 36 and d 72) were analyzed to determine concentrations of glucose, albumin, TP and urea nitrogen (BUN) using commercial kits (Pars Azmoon Co., Tehran, Iran). Further, serum beta-hydroxybutyrate (BHB) (Abbott Diabetes Care Ltd., Oxin, UK), aspartate aminotransferase (AST), alanine aminotransferase (ALT), and insulin concentration were analyzed using Auto Analyzer (Hitachi 717, Japan). The inter-and intra-assay CV% were 3.76 and 5.06 for BHB and 2.96 and 5.42 for insulin, respectively.

### 2.7. Statistical Analysis

The MIXED procedure of SAS was used with including the fixed effects of time, grain source, starter CP content, and their interactions and random effect of calf using the following model with repeated measures over time: Yijkl = μ + Gi + CPj + Tk + (G × T)ik + (CP × T)jk + (G × CP)ij + (G × CP × T)ijk + β(Xi - X) + εijkl where Yijk is the dependent variable; µ is the overall mean; Gi is the effect of grain source (CG vs. BG); CPj is the effect of starter CP content (19% vs. 22); Tk is the effect of time; (G × CP)ij is the interaction between grain source and time; (CP × T)ik the interaction between starter CP content and time; (G × CP)jk is the interaction between grain source and starter CP content; (G × CP × T)ijk is the tripartite effect of grain source, starter CP content, and time; β(Xi − X) is the covariate variable (used BW and growth indices with initial BW and growth indices as covariate); and εijk is the overall error term. Two variance-covariance structures, i.e., auto-regressive type 1 and compound symmetry, were tested and the covariance structure with the smallest Schwarz’s Bayesian information criterion was chosen. The threshold of significance was set at *p* ≤ 0.05; trends were declared at 0.05 < *p* ≤ 0.10.

## 3. Results

### 3.1. Performance and Nutrient Digestibility

Starter and total DMI intakes were similar across treatments during all 3 periods (*p* > 0.05; Table 2). The ADG tended to be higher (*p* = 0.09) and was higher (*p* = 0.03) in BG calves compared with CG groups during the pre- and post-weaning periods, respectively. The weaning BW tended to be greater (*p* = 0.08) and final BW was greater in BG than CG calves (*p* = 0.05). Further, feed efficiency improved in calves who received BG based starter feed compared with CG groups during the pre-weaning (*p* = 0.04), post-weaning (*p* = 0.02) and overall (*p* = 0.03) periods. Starter protein content had no effect on intake and growth rate; however, FE tended to be greater in 22CP groups than 19CP calves during the post-weaning period (*p* = 0.06). In general, no significant interaction (*p* > 0.05) was detected between grain source and protein content of starter feed for intake, weight gain and FE. DM, CP, and ether extract digestibility did not differ among treatments; however, NDF digestibility was increased (*p* = 0.03) when calves received starter with 22CP compared with 19CP groups. 

### 3.2. Skeletal Growth Parameters

Heart girth, body length, body girth, and hip width were not affected by treatments (*p* > 0.05) (Table 3). However, hip height tended (*p* = 0.09) to be greater in BG calves compared to calves received CG. Moreover, hip height was greater in 22CP calves compared with 19CP groups at weaning (*p* = 0.02) and was greater (*p* = 0.05) at final measurements. The greatest wither height was observed for BG-22CP at final measurement (*p* = 0.03). 

### 3.3. Ruminal Fermentation Profile

Ruminal pH was not affected by treatments (*p* > 0.05; Table 4). Ruminal NH_3_-N concentration was higher (*p* = 0.02) when calves fed CG compared with BG on d 71. Further, ruminal NH_3_-N concentration was greater (*p* = 0.05) in dairy calves fed higher dietary protein content on d 71, regardless of grain source. The ruminal concentration of SCFA was greater in barley-based diet than corn-based diet at d 35 and 71 (*p* = 0.01 and *p* = 0.03, respectively). At d 35, the molar proportion of acetate was increased (*p* = 0.02) in CG calves compared with BG groups, but the molar proportions of propionate and butyrate in BG calves was greater than CG groups. The acetate: propionate ratio was greater in animals fed on CG based-diet (*p* = 0.01) than BG calves on d 35. The concentration of valerate (*p* = 0.05) in the ruminal fluid was increased in the pre-weaning period when calves fed greater dietary protein content. 

### 3.4. Blood Metabolites, Liver Enzymes, and Insulin

The blood glucose, total protein, and albumin concentrations did not differ among treatments (Table 5). However, BHB concentration was increased in calves fed on BG (*p* < 0.05) compared with CG calves on days 36 and 72. Moreover, calves received 22CP diets had greater BHB than those fed 19CP on d 36 (*p* = 0.02). The calves fed 22CP diets had greater BUN than 19CP groups on d 72 (*p* = 0.02), regardless of grain source. Further, the BUN concentration was reduced in BG calves compared with CG calves on d 72 (*p* = 0.03). Liver enzymes concentrations were similar (*p* > 0.05) across treatments in the current study. At d 36, the blood insulin concentration was increased (*p* = 0.04) when calves fed on BG, regardless of dietary protein content. The greatest blood insulin concentration was observed for BG-22CP among treatments (*p* = 0.03). 

## 4. Discussion

### 4.1. Grain Source Effect

Controversial results have been reported regarding the effects of different grain sources on the intake in dairy calves [3,11,30]. Different processing methods [3,15] could significantly influence the intake through ruminal pH alterations. Khan et al. [3] reported that lower ruminal pH in the calves fed on BG depressed intake. Different particle size, [11] as well as starch availability rate [10] could influence ruminal pH. However, similar ruminal pH across experimental diets were observed in the current study, which was partly because GMPS was similar among experimental diets (Table 1). The calves fed with BG diets had greater BW and subsequently greater FE (0.439 vs. 0.517 for CG and BG, respectively) at the end of the experiment. Total SCFA concentration in the ruminal fluid of BG calves was greater compared with CG calves both in pre- and in post-weaning periods. This is in line with findings on dairy cows that BG produced higher SCFA compared to CG [9]. It is notable that a high concentration of ruminal SCFA (>150 mM/L) can induce ruminal acidosis [31]. However, the range of the ruminal SCFA concentration in the current study was in the range of previous studies [3,4,11]. Corn starch is less available for ruminal fermentation than barley grain and, hence, there is a need to conduct more extensive processing, such as steam-flaking instead of a simple grinding, to achieve higher availability of starch [1,12]. The main energy source for ruminants is SCFA that could be an indicator of the ruminal fermentation [32]. Therefore, the greater SCFA observed in BG diets, the higher the “whole body” efficiency towards greater gain and productivity. Among individual SCFA the molar proportions of propionate and butyrate were increased in calves fed BG, and in contrast, acetate was in a lower range in this diet. Propionate and butyrate are two main ruminal volatile fatty acids that function as chemical stimuli for rumen development and they could potentially supply greater energy than acetate [32].

Lower ruminal NH_3_-N concentration in BG calves in comparison with CG diets could be an indicator of greater N utilization in calves offered with barley. Accordingly, BUN concentration was lower in BG calves as well. Our results suggest that BG was more beneficial in N capturing and reducing both ruminal NH_3_-N and BUN concentrations compared to CG. A linear decrease in ruminal NH_3_-N concentration in dairy cows was reported as a result of an increasing proportion of BG in the diet [9]. This may be related to the BG starch structure which is rapidly degradable and easier accessible for ruminal microbes [9,10]. DePeters and Fergusen [33] reported that BUN could be used as an indicator of ruminal N captures, as this value is positively associated with ruminal NH_3_-N concentration. Further, the BUN is a useful indicator of N efficiency status. A linear relationship between BUN and urinary N excretion was previously reported [34]. Our result is in line with previous studies that showed processing of high-starch grains could influence the N utilization efficiency in dairy calves that is mostly due to higher accessibility of starch in processed grains for ruminal microbes [4,19,20]. 

Higher blood BHB concentration in BG calves during the pre-weaning period is mostly related to greater butyrate concentration in the ruminal fluid of calves fed BG. Although the glucose concentration was similar among treatments, insulin concentration was increased in BG diets. Although glucose concentration is the main regulator of blood insulin concentration [10], the greater SCFA concentration could stimulate insulin secretion in ruminants as well [35]. Sakata et al. [36] suggested that the stimulatory effect of administration of SCFA such as propionate and butyrate into the rumen upon the mitotic index of rumen epithelial cells might be due to an increase in plasma insulin concentration. Higher blood insulin concentration can be also due to increased AA uptake which has been previously proposed in growing lambs [37] and growing calves [38]. 

### 4.2. Effect of Starter Protein Level

The intake and performance were maximal for calves fed a 19.6% CP starter when starter diets contained 15% to 22.4% CP [16]. The results of the current study suggest that starter protein content was not as effective as other studies in influencing starter intake, gain, and efficiency [16]. In addition to the starter protein content, starter feeding strategy might also be a factor influencing the animal response to dietary protein content. For instance, Stamey et al. [39] reported that starter with 25.5% CP (DM basis) provided modest benefits in starter intake (particularly around weaning) and growth for dairy calves in an enhanced early nutrition program compared with a conventional starter diet contained 19.6% CP. Regarding the growth indices, the 22CP diets improved hip height compared to 19CP diets. According to previous studies, greater AA content may support increased growth in growing animals [40,41]. 

Digestibility of NDF was improved when a starter diet with greater protein content was offered to calves. Soybean meal is a protein source that could provide relatively high ruminal peptide concentration after degradation and improve fiber digestion [42]. Moreover, the greater valerate concentration which was observed in 22CP diets and originated from the higher AAs, provided by 22PC diets, could positively influence the fiber digestion [42]. Irrespective of the CP content of the starter diet, the improvement in NDF digestibility may primarily be related to higher intrinsic NDF digestibility of fiber supplied though SBM. In our study, dry matter digestibility did not change across experimental treatments.

The BUN concentration was lower for 19CP compared to 22CP diets. This was attributed to excess N concentrations in the rumen fluid of post-weaning calves fed with 22CP diets. This is probably due to lower protein requirements of weaned compared to pre-weaning calves [40]. 

### 4.3. Interaction of Grain Source and Starter Protein Content

Although ADG and FE were influenced by grain source, no interaction was found for the grain source with starter protein content. The greatest wither height was observed for BG-22CP (92.9 cm). Our results suggest that feeding calves with BG with greater starter protein content could stimulate dairy calf growth. This could, probably, be due to higher availability of starch provided by BG along with higher available N provided by 22CP diets in the rumen. This, probably, is related to well-matched fermentation of starch supplied by BG rather than CG with higher starter protein content [4]. Furthermore, the greatest blood insulin concentration was observed in BG-22CP treatment in the current study (11.7 IU/L). As discussed earlier, greater blood insulin concentration may be related with greater growth indices in calves fed with BG and higher starter protein content. The importance of wither height of female calves under 6 months of age on production traits in first calving has been well documented [41]. Furthermore, Heinrichs and Hargrove [43] observed that the correlation between wither height and first-lactation milk yield was greater (0.41) than that of BW (0.34) in dairy calf heifers. 

## 5. Conclusions

Here we provide evidence that feeding dairy calves with BG as a sole grain source in starter diets increases growth rate, feed efficiency, and efficient ruminal metabolism in comparison with CG. Higher starter protein content improved fiber digestibility and growth indices in dairy calves. Feeding calves with starters containing BG and higher protein content (i.e., 22% CP) improved the wither height which could be advantageous in rearing programs of the replacement heifers. The higher concentrations of blood insulin, ruminal SCFA, and nitrogen efficiency may contribute to improved animal growth performance found in BG-22CP diet. 

## Figures and Tables

**Table 1:** Experimental starter diet ingredients, chemical composition (g/kg of DM, unless otherwise stated) and particle size distribution [% mean ± SD] of experimental diets.

Item	Treatments ^1^			
	CG		BG	
	19CP	22CP	19CP	22CP
**Ingredients**				
Alfalfa hay, chopped	50	50	50	50
Corn grain, ground	586	506	0	0
Barley grain, ground	0	0	626	536
Soybean meal	240	320	200	290
Wheat bran	40	40	40	40
Fat source	15	15	15	15
Molasses, beet pulp	15	15	15	15
Carbonate calcium	11.5	11.5	11.5	11.5
Di-calcium phosphate	7.5	7.5	7.5	7.5
Sodium bicarbonate	10	10	10	10
White salt	5	5	5	5
Vitamin and mineral mix ^2^	20	20	20	20
**Chemical composition**				
Metabolisable energy ^3^ (Mcal/kg)	2.85	2.84	2.82	2.81
Crude protein ^5^	190	220	190	220
Rumen undegradable protein, % of CP ^3^	28.9	27.4	26.3	26.1
Non-fiber carbohydrate ^4^	546	511	503	473
Neutral detergent fiber ^5^	160	164	195	190
NDF: NFC	0.29	0.32	0.38	0.40
Starch ^6^	361	333	335	295
Ether extract ^5^	43	43	40	41
Calcium ^5^	8	8	8	8
Phosphorus ^5^	4.7	4.7	4.7	4.7
**Experimental diets particle size distribution**				
4.75, mm	1.3 ± 0.06	1.4 ± 0.01	1.4 ± 0.03	1.3 ± 0.02
0.6, mm	34.6 ± 0.32	35.5 ± 0.44	35.4 ± 0.42	34.8 ± 0.10
0.3, mm	15.7 ± 0.14	13.9 ± 0.19	17.1 ± 0.12	15.4 ± 0.17
0.15, mm	12.2 ± 0.07	11.3 ± 0.12	13.0 ± 0.18	12.9 ± 0.19
Pan	10.5 ± 0.14	12.7 ± 0.10	10.7 ± 0.20	10.0 ± 0.22
Geometric mean particle size	0.61 ± 0.02	0.63 ± 0.01	0.60 ± 0.02	0.61 ± 0.01

^1^ Treatments were; corn grain based starter diet with 19% crude protein (corn grain (CG)-19CP); corn grain based starter diet with 22% crude protein (CG-22CP); barley grain based starter diet with 19% crude protein (barley grain (BG)-19CP); and barley grain based starter diet with 22% crude protein (BG-22CP). ^2^ Contained per kilogram of supplement: 800,000 IU vitamin A, 150,000 IU vitamin D, 1500 IU vitamin E, 100 g Ca, 2g Zn, 60 g *p*, 12 g Mg, 10 g Na, 2 g Fe, 50 mg Co, 200 mg Cu, 40 mg I, 1g Mn, and 2 mg Se. ^3^ Calculated from NRC (2001).^4^ Non-fiber-carbohydrate was calculated as [DM- (NDF + CP + ether extract + ash)] (NRC, 2001).^5^ Values were chemically analyzed in laboratory.^6^ Calculated by Cornell Net Carbohydrate and Protein System models (CNCPS, v. 6.1).

**Table 2:** Least square means for starter intake, average daily gain, feed efficiency, and nutrient digestibility in dairy calves fed different grain sources (corn grain vs. barley grain) and different protein levels (19% vs. 22 % of DM) (*n* = 10 calves per treatment).

Item	Treatments ^1^				SEM	*p-*Value ^2^		
	CG		BG			GS	CP	GS × CP
	19CP	22CP	19CP	22CP				
**Starter feed intake, g/d**							
Pre-weaning (d 3–59)	430	434	388	368	43.0	0.21	0.86	0.78
Post-weaning (d 59–73)	1975	1981	2110	1852	161.5	0.98	0.43	0.41
Entire period (d 3–73)	650	656	634	583	80.4	0.56	0.76	0.70
Milk intake, g/d	632	630	632	631	2.19	0.91	0.90	0.93
**Total dry matter intake (milk + starter), g/d**								
Pre-weaning (d 3–59)	1063	1067	1020	1004	48.10	0.27	0.89	0.83
Entire period (d 3–73)	1195	1199	1178	1127	61.75	0.46	0.70	0.65
**Average daily gain, g/d**								
Pre-weaning (d 3–59)	490	496	541	560	47.98	0.09	0.72	0.88
Post-weaning (d 59–73)	705	736	810	917	81.58	0.03	0.41	0.66
Entire period (d 3–73)	521	530	580	611	43.83	0.08	0.64	0.80
**Body weight, kg**								
Initial (d 3)	39.3	39.1	39.2	39.3	1.06	0.93	0.96	0.86
Weaning (d 59)	68.9	69.0	71.6	72.3	1.60	0.08	0.86	0.90
Final (d 73)	75.9	76.3	79.7	81.4	1.89	0.05	0.66	0.79
**Feed efficiency ^3^, g daily gain/g daily intake**								
Pre-weaning (d 3–59)	0.456	0.462	0.529	0.557	0.03	0.04	0.70	0.89
Post-weaning (d 59–73)	0.358	0.372	0.385	0.496	0.04	0.02	0.06	0.25
Entire period (d 3–73)	0.436	0.443	0.493	0.541	0.03	0.03	0.67	0.91
**Nutrients apparent digestibility, g/kg**								
Dry matter	699	692	698	701	14.7	0.73	0.85	0.62
Crude protein	718	740	743	752	24.8	0.33	0.39	0.79
Neutral detergent fiber	605	637	613	649	18.6	0.50	0.03	0.91
Ether extract	766	769	790	779	23.6	0.35	0.84	0.69

^1^ Treatments were; corn grain-based starter diet with 19% crude protein (CG-19CP); corn grain-based starter diet with 22% crude protein (CG-22CP); barley grain-based starter diet with 19% crude protein (BG-19CP); and barley grain-based starter diet with 22% crude protein (BG-22CP). ^2^ Statistical comparisons: GS = grain sources (corn grain vs. barley grain); CP = 19% vs. 22% crude protein based on dry matter intake; and GS × CP = interaction of grain source and starter dietary protein level. ^3^ kg of body weight gain/kg of total dry matter intake.

**Table 3:** Least square means for growth parameters in dairy calves fed different grain sources (corn grain vs. barley grain) and different protein levels (19% vs. 22 % of DM) (*n* = 10 calves per treatment).

Item	Treatments ^1^				SEM	*p-*Value ^2^		
	CG		BG			GS	CP	GS × CP
	19CP	22CP	19CP	22CP				
**Heart girth, cm**								
Initial (d 3)	81.9	81.6	81.3	80.9	0.99	0.51	0.72	0.96
Weaning (d 59)	98.3	98.7	99.5	99.4	1.05	0.37	0.87	0.83
Final (d 73)	99.3	100.2	101.5	100.6	1.20	0.28	0.97	0.48
**Body length, cm**								
Initial (d 3)	44.4	45.9	46.8	46.4	1.02	0.16	0.59	0.36
Weaning (d 59)	57.0	55.8	55.7	55.9	1.34	0.60	0.66	0.54
Final (d 73)	61.1	60.3	59.4	60.2	1.09	0.42	0.97	0.47
**Body girth, cm**								
Initial (d 3)	84.8	84.7	83.4	83.1	1.20	0.22	0.86	0.93
Weaning (d 59)	109.5	110.5	108.0	109.1	1.54	0.36	0.50	0.97
Final (d 73)	114.9	114.7	114.5	116.2	1.79	0.76	0.67	0.60
**Wither height, cm**								
Initial (d 3)	78.7	79.7	78.0	78.9	0.85	0.38	0.27	0.95
Weaning (d 59)	90.3	88.4	89.7	90.8	0.64	0.20	0.41	0.02
Final (d 73)	91.8	90.2	91.7	92.9	0.71	0.04	0.54	0.03
**Hip height, cm**								
Initial (d 3)	81.8	81.0	80.6	81.7	0.91	0.74	0.91	0.28
Weaning (d 59)	93.2	95.1	93.4	95.2	0.78	0.83	0.02	0.92
Final (d 73)	94.3	95.8	95.6	97.0	0.72	0.09	0.05	0.94
**Hip width, cm**								
Initial (d 3)	19.7	20.7	20.0	20.2	0.27	0.80	0.13	0.31
Weaning (d 59)	21.2	21.3	21.0	21.8	0.46	0.68	0.30	0.41
Final (d 73)	22.1	22.2	22.2	22.5	0.36	0.54	0.55	0.73

^1^ Treatments were; corn grain-based starter diet with 19% crude protein (CG-19CP); corn grain-based starter diet with 22% crude protein (CG-22CP); barley grain-based starter diet with 19% crude protein (BG-19CP); and barley grain-based starter diet with 22% crude protein (BG-22CP). ^2^ Statistical comparisons: GS = grain sources (corn grain vs. barley grain); CP = 19% vs. 22% crude protein based on dry matter intake; and GS × CP = interaction of grain source and starter dietary protein level.

**Table 4:** Least square means for ruminal metabolism in dairy calves fed different grain sources (corn grain vs. barley grain) and different protein levels (19% vs. 22 % of DM) (*n* = 10 calves per treatment).

Item	Treatments ^1^				SEM	*p-*Value ^2^		
	CG		BG			GS	CP	GS × CP
	19CP	22CP	19CP	22CP				
**Ruminal pH**								
d 35	5.6	5.6	5.6	5.5	0.08	0.23	0.70	0.63
d 71	5.8	5.9	5.8	5.8	0.04	0.59	0.66	0.37
**Ruminal NH_3_-N, mg/dL**								
d 35	9.8	11.1	9.1	9.4	0.45	0.08	0.23	0.45
d 71	13.2	14.6	11.1	12.1	0.71	0.02	0.05	0.28
**Total short-chain fatty acids, mM/L**								
d 35	83	89	99	102	3.34	0.01	0.16	0.56
d 71	106	109	113	121	2.85	0.03	0.19	0.16
**Individual short chain fatty acids, mM**								
**Acetate (A)**								
d 35	54.0	50.9	49.1	46.2	1.47	0.02	0.06	0.92
d 71	53.6	52.9	53.1	50.3	1.02	0.13	0.17	0.23
**Propionate (P)**								
d 35	29.2	30.3	31.7	33.4	1.11	0.03	0.14	0.98
d 71	32.7	33.4	34.8	36.3	1.08	0.09	0.49	0.79
**A: P**								
d 35	1.87	1.66	1.55	1.38	0.09	0.01	0.07	0.81
d 71	1.65	1.60	1.53	1.41	0.05	0.06	0.30	0.68
**Butyrate**								
d 35	11.8	12.2	15.1	14.9	1.25	0.04	0.93	0.79
d 71	8.7	9.0	7.9	8.9	1.22	0.80	0.72	0.84
**Valerate**								
d 35	3.9	4.7	3.2	4.6	0.55	0.47	0.05	0.57
d 71	4.0	3.2	3.3	3.6	0.28	0.75	0.48	0.18
**Isovalerate**								
d 35	0.86	1.04	0.83	0.79	0.29	0.65	0.81	0.71
d 71	0.75	0.83	0.51	0.72	0.15	0.53	0.51	0.89

^1^ Treatments were; corn grain-based starter diet with 19% crude protein (CG-19CP); corn grain-based starter diet with 22% crude protein (CG-22CP); barley grain-based starter diet with 19% crude protein (BG-19CP); and barley grain-based starter diet with 22% crude protein (BG-22CP). ^2^ Statistical comparisons: GS = grain sources (corn grain vs. barley grain); CP = 19% vs. 22% crude protein based on dry matter intake; and GS × CP = interaction of grain source and starter dietary protein level.

**Table 5:** Least square means for blood metabolites, liver enzymes, and insulin in dairy calves fed different grain sources (corn grain vs. barley grain) and different protein levels (19% vs. 22 % of DM) (*n* = 10 calves per treatment).

Item	Treatments ^1^				SEM	*p-*Value ^2^		
	CG		BG			GS	CP	GS × CP
	19CP	22CP	19CP	22CP				
**Glucose, mg/dL**								
d 36	111	108	113	112	5.42	0.47	0.63	0.84
d 72	83	82	80	81	4.18	0.58	0.96	0.77
**Beta-hydroxybutyrate, mM**								
d 36	0.09	0.10	0.10	0.13	0.009	0.01	0.02	0.46
d 72	0.22	0.23	0.31	0.29	0.03	0.03	0.89	0.61
**Total protein, mg/L**								
d 36	5.9	6.1	5.7	5.9	0.15	0.22	0.12	0.93
d 72	6.1	6.6	6.3	6.5	0.25	0.70	0.14	0.56
**Albumin, mg/L**								
d 36	3.2	3.2	3.1	3.2	0.05	0.97	0.44	0.53
d 72	3.1	3.1	3.1	3.2	0.07	0.76	0.27	0.75
**Blood urea nitrogen, mg/dL**								
d 36	13.7	16.2	13.7	15.5	1.06	0.31	0.23	0.34
d 72	21.2	26.0	17.7	21.9	1.99	0.03	0.02	0.63
**Aspartate aminotransferase, IU/L**								
d 36	39.5	41.9	42.7	37.9	3.07	0.90	0.69	0.25
d 72	49.5	67.8	61.7	61.8	8.10	0.76	0.38	0.39
**Alanine aminotransferase, IU/L**								
d 36	8.7	9.0	10.1	8.5	0.60	0.46	0.29	0.12
d 72	13.1	13.8	13.0	13.1	1.51	0.75	0.81	0.82
**Insulin, IU/l**								
d 36	5.6	6.6	6.7	9.9	0.81	0.04	0.06	0.33
d 72	9.6	7.2	8.5	11.7	0.93	0.06	0.66	0.03

^1^ Treatments were; corn grain-based starter diet with 19% crude protein (CG-19CP); corn grain-based starter diet with 22% crude protein (CG-22CP); barley grain-based starter diet with 19% crude protein (BG-19CP); barley grain-based starter diet with 22% crude protein (BG-22CP). ^2^ Statistical comparisons: GS = grain sources (corn grain vs. barley grain); CP = 19% vs. 22% crude protein based on dry matter intake; and GS × CP = interaction of grain source and starter dietary protein level.
